# Supplementary material for: Imaging quantized vortex rings in superfluid helium to evaluate quantum dissipation
Source: Nat Commun. 2023 May 23;14:2941. doi: 10.1038/s41467-023-38787-w (PMC10205744; doi:10.1038/s41467-023-38787-w)
Supplement: Supplementary file 1 — Supplementary Information [file 41467_2023_38787_MOESM1_ESM.pdf]

Supplementary Information: Imaging quantized vortex rings in superfluid helium to evaluate quantum dissipation

Yuan Tang,[1,2] Wei Guo,[1,2,*] Hiromichi Kobayashi,[3,4]
Satoshi Yui,[5,6] Makoto Tsubota,[5,6] and Toshiaki Kanai[1,7]

[1]*National High Magnetic Field Laboratory, 1800 East Paul Dirac Drive, Tallahassee, FL 32310, USA*

[2]*Mechanical Engineering Department, FAMU-FSU College of Engineering, Florida State University, Tallahassee, FL 32310, USA*

[3]*Research and Education Center for Natural Sciences, Keio University, 4-1-1 Hiyoshi, Kohoku-ku, Yokohama 223-8521, Japan*

[4]*Department of Physics, Hiyoshi Campus, Keio University, 4-1-1 Hiyoshi, Kohoku-ku, Yokohama 223-8521, Japan*

[5]*Department of Physics, Osaka Metropolitan University, 3-3-138 Sugimoto, Sumiyoshi-ku, Osaka 558-8585, Japan*

[6]*Nambu Yoichiro Institute of Theoretical and Experimental Physics (NITEP), Osaka Metropolitan University, Osaka 558-8585, Japan*

[7]*Department of Physics, Florida State University, Tallahassee, FL 32306, USA*

* Email: wguo@magnet.fsu.edu

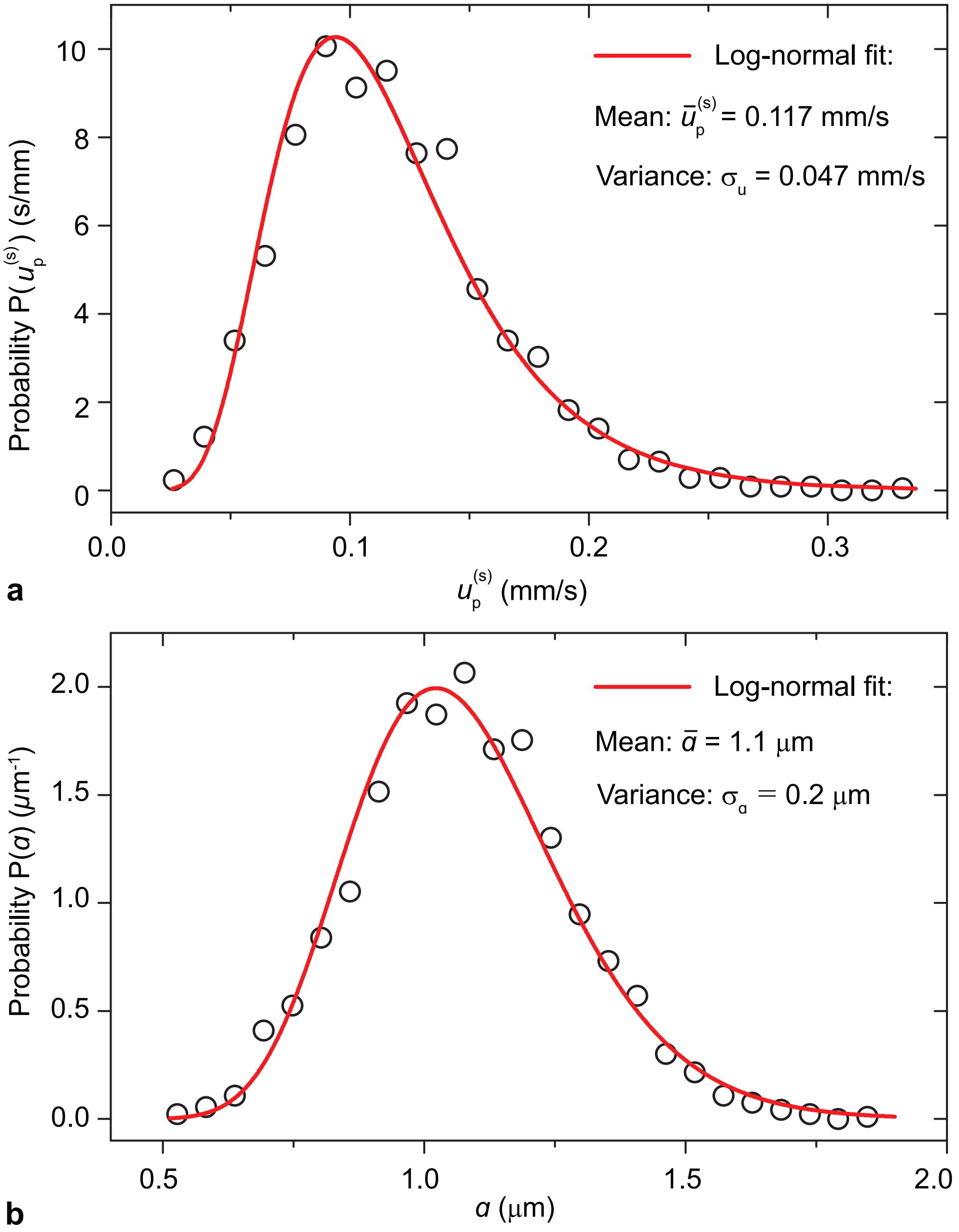

Supplementary Fig. 1. Settling velocity and radius distribution of the D_2 particles in He II at T=1.65 K. a, Distribution of the settling velocity $u_p^{(s)}$. **b**, Distribution of the particle radius a.

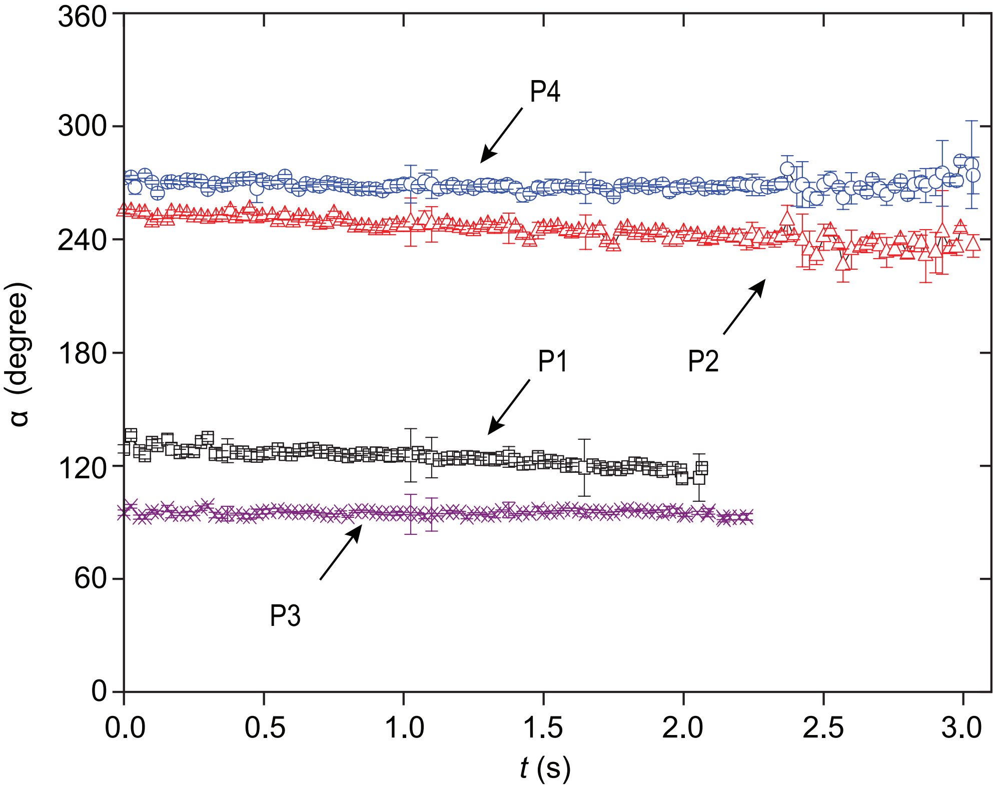

Supplementary Fig. 2. **Time evolution of the angular position** α **of representative trapped particles for the 9-particle ring event.** The definition of the angle α is given in the main text. The error bars denote the standard deviation associated with the data due to the uncertainties of the particle positions.

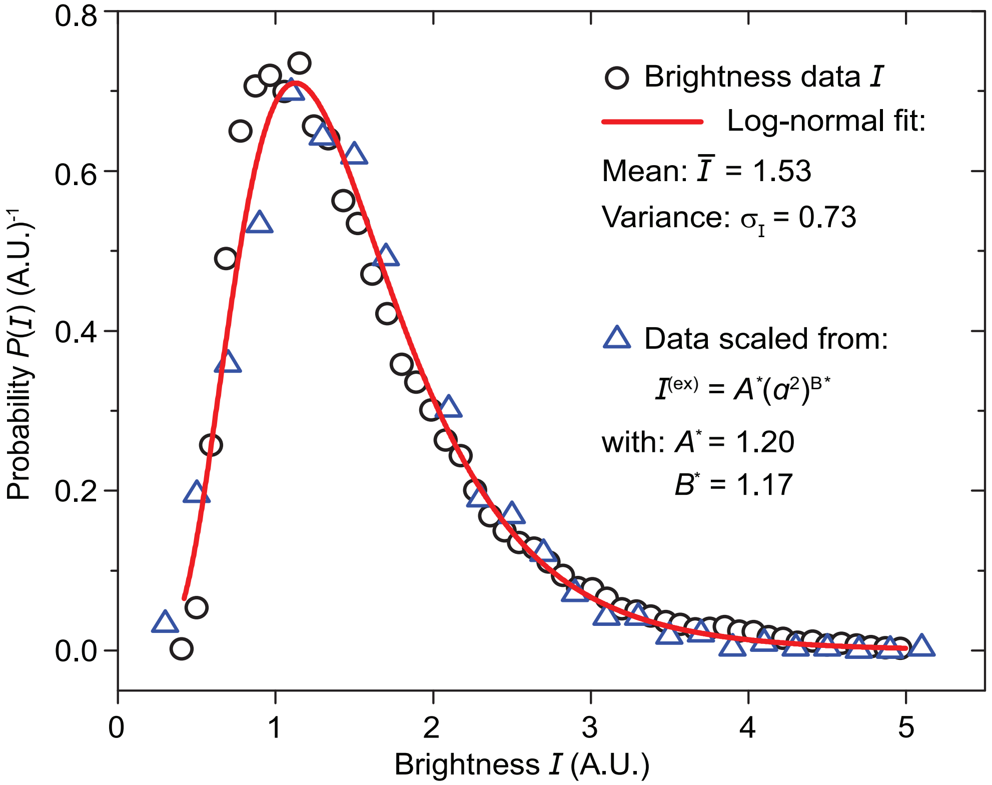

Supplementary Fig. 3. **Distribution of the brightness** I **of the** D_2 **particles.** The black circles represent the measured brightness I. The blue triangles are $I^{(ex)}$ calculated using the distribution of particle radius a, where $A^* = 1.20$ and $B^* = 1.17$ are the optimal correlation parameters that render the best agreement between the two distributions.

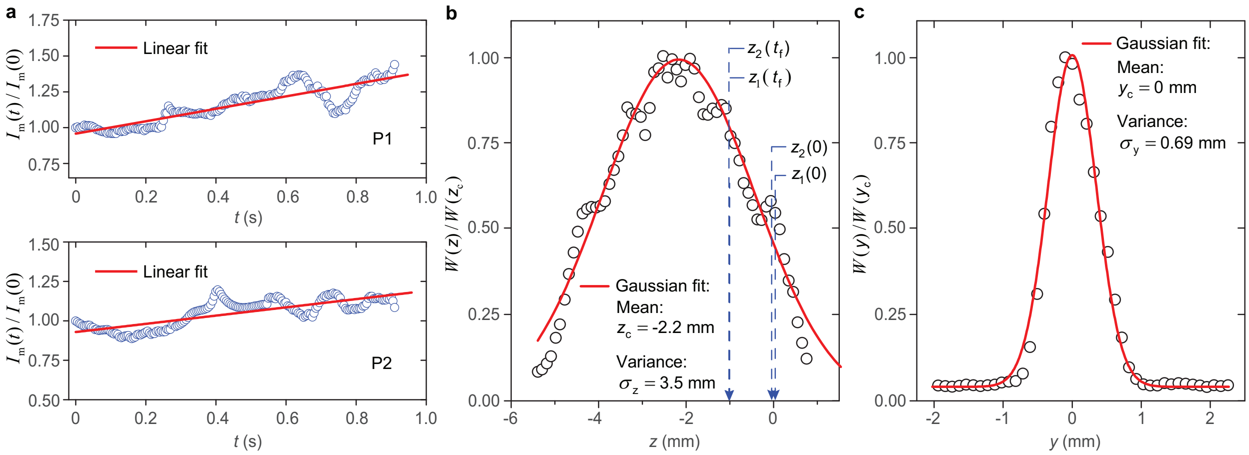

Supplementary Fig. 4. Trapped particle's brightness variation and laser-intensity cross-sectional profile. a, Time variation of the directly measured brightness $I_{\mathrm{m}}(t)$ of the two trapped particles as shown in **Fig.2a**. **b**, Measured laser intensity W as a function of z (i.e., height direction). The red curve is a Gaussian fit to the data. The z-coordinates of the two particles at $t = 0$ and $t = t_{\mathrm{f}}$ are indicated. **c**, Measured laser intensity W as a function of y (i.e., thickness direction).

Supplementary Table. 1. Radiuses and initial positions of the trapped particles for the 9-particle vortex ring in **Fig.1** and the 2-particle vortex ring in **Fig.2**.

9-p ring	x (mm)	Δx (mm)	y (mm)	Δy (mm)	z (mm)	Δz (mm)	a (μm)	Δa (μm)
P1	-0.271	0.003	-0.209	0.005	0.204	0.002	0.87	0.18
P2	-0.186	0.002	0.261	0.004	-0.074	0.002	1.32	0.53
P3	-0.101	0.003	-0.269	0.005	0.220	0.002	1.04	0.30
P4	-0.093	0.002	0.270	0.004	-0.089	0.002	1.69	0.38
P5	-0.008	0.002	0.254	0.003	-0.089	0.002	1.74	0.33
P6	0.154	0.003	0.155	0.004	-0.051	0.002	1.03	0.37
P7	0.215	0.002	0.027	0.003	0.016	0.002	0.92	0.26
P8	-0.358	0.002	0.146	0.005	0.011	0.003	0.78	0.19
P9	0.216	0.002	-0.046	0.003	0.058	0.002	1.09	0.24
2-p ring	x (mm)	Δx (mm)	y (mm)	Δy (mm)	z (mm)	Δz (mm)	a (μm)	Δa (μm)
P1	-0.008	0.005	–	–	0.047	0.004	1.18	0.08
P2	0.271	0.004	–	–	-0.047	0.004	1.12	0.07
