# Supplementary material for: Imaging quantized vortex rings in superfluid helium to evaluate quantum dissipation
Source: Nat Commun. 2023 May 23;14:2941. doi: 10.1038/s41467-023-38787-w (PMC10205744; doi:10.1038/s41467-023-38787-w)
Supplement: Supplementary file 3 — Description of Additional Supplementary Files [file 41467_2023_38787_MOESM3_ESM.pdf]

File name: Supplementary Movie 1

Description: A movie consisting of a few short video clips showing the propagation of representative quantized vortex rings in superfluid helium-4. These quantized vortex rings are visualized by imaging the trapped D₂ tracer particles.

File name: Supplementary Movie 2

Description: A movie consisting of a few short video clips showing how quantized vortex rings are created as a consequence of reconnections of intersecting quantized vortex lines in superfluid helium-4.

File name: Supplementary Movie 3

Description: A movie showing the motion of a quantized vortex ring which carries nine discrete D₂ tracer particles moving through quiescent superfluid helium-4. This event is analyzed and presented in Fig.1 in the paper.

File name: Supplementary Movie 4

Description: A movie consisting of three short video clips showing three representative vortex-ring events where only two D₂ tracer particles are trapped on the vortex ring. For these events, the background flow of superfluid helium-4 is negligible. The analysis results of the first 2-particle vortex-ring event is presented in Fig. 2 in the paper.

File name: Supplementary Movie 5

Description: A movie consisting of a few short video clips showing representative vortex-ring events where the rings heavily doped with D₂ tracer particles spontaneously flip to the downward direction while they propagate and shrink in superfluid helium-4. The analysis of the first event is presented in Fig.3 in the paper.

File name: Supplementary Movie 6

Description: A movie consisting of a few short video clips showing representative vortex ring events where the trapped D₂ tracer particles detach from the vortex core due to the Stokes drag from the normal fluid, as the ring diminishes. The detached particles then aggregate to form a cluster that slowly falls in superfluid helium-4. The analysis of the first event is presented in Fig.3 in the paper.

File name: Supplementary Movie 7

Description: A representative movie showing the settling motion of D₂ tracer particles in quiescent superfluid helium-4. This movie is taken in the same experiment run as the 9-particle ring event and the 2-particle ring event presented in the paper.
